# The Development of High-Quality Multispecies Probiotic Formulations: From Bench to Market

**DOI:** 10.3390/nu12082453

**Published:** 2020-08-15

**Authors:** Lukas Grumet, Yorick Tromp, Verena Stiegelbauer

**Affiliations:** 1 Institut AllergoSan, Gmeinstraße 13, 8055 Graz, Austria; info@allergosan.at; 2 Winclove Probiotics, Hulstweg 11, 1032 LB Amsterdam, The Netherlands; y.tromp@winclove.nl

**Keywords:** probiotics, multispecies, quality criteria, safety aspects, clinical evidence

## Abstract

Probiotics are live microorganisms that, when administered in adequate amounts, confer a health benefit on the host. To date, there is an increasing number of commercially available products containing probiotics on the market. Probiotics have been recommended by health care professionals for reasons ranging from their long-term immunomodulatory effects to proven benefits in the management of different health conditions. For probiotic products, there are several important aspects that determine the success rate of the development from bench to market. The aim of this review is to explore how the current knowledge on microbe–microbe and host–microbe interactions can be used to develop high-quality, evidence-based probiotic formulations, specifically probiotic dietary supplements, with a focus on the selection of safe strains with relevant functional properties. In addition, we will highlight aspects of the probiotic manufacturing process that need to be considered during the product development and the subsequent manufacturing process to guarantee consistent efficacy of a probiotic product. For each high-quality probiotic formulation, it is important to screen multiple strains, and select only those strains that show relevant functional properties and that can be considered safe for human consumption. In addition, it is imperative that attention is paid to the product development and manufacturing process, and that safety and quality properties are monitored. Importantly, the beneficial effects of probiotics should be evaluated in product efficacy studies and post-marketing surveys in order to demonstrate their clinical efficacy. All these aspects need to be evaluated and validated during the development of a successful high-quality and ready-to-market probiotic.

## 1. Introduction: Probiotics and the Role of Microorganisms in Human Health

The human body is populated by a vast array of microorganisms and most of them co-exist in or on the human body without causing harm to their host. In recent decades, we have started to appreciate these microorganisms for more than just their contribution to the digestion of our food. Although we are just barely beginning to understand the vastness of the complex networks of microbe–microbe and host–microbe interactions that take place on every bodily surface, we do acknowledge that all these interactions are tremendously important for our health [1]. Since our body is not able to perform all vital biochemical reactions required for health homeostasis by itself, we depend on the presence and activities of commensal microorganisms. Advancements in biotechnology and medicine enabled us to not only grow but also thoroughly investigate bacteria that harbor potentially healthy properties. Such microorganisms can be used to specifically support or restore homeostasis and contribute to our health; in this case, we refer to these microorganisms as probiotics. Probiotics are live microorganisms that, when administered in adequate amounts, confer a health benefit on the host [2]. The most widely used microorganisms that exhibit probiotic properties and that are included in probiotic products such as functional foods and dietary supplements are *Bifidobacterium* spp. and lactic acid bacteria.

In recent decades, a large number of health conditions (diseases, disorders, syndromes and afflictions) have been associated with changes in microbiota composition and activity (now commonly referred to as dysbiosis). For example, scientific evidence links an imbalance of the intestinal microbiota to a growing number of diseases or syndromes, such as inflammatory bowel disease (IBD), irritable bowel syndrome (IBS), acute, nosocomial, and antibiotic-associated diarrhea (AAD), allergic disorders such as atopic dermatitis (eczema) and allergic rhinitis, colorectal cancer, and metabolic disorders such as obesity, metabolic syndrome, non-alcoholic fatty liver disease, and type 2 diabetes [3,4]. Similarly, imbalances in, for example, the vaginal microbiota have been associated with vaginal infections. Most of this research is still indirect or associative, which means that it is not clear whether microbiota imbalances are a cause or a consequence of the respective disease. However, the intimate relation between microbiota and human disease is suggested by many publications, and the evidence of cause-and-effect studies is steadily increasing [5]. For most health conditions, probiotics have been studied for their potential to prevent and/or ameliorate the respective condition or for their potential as coadjuvants in the treatment of certain conditions (i.e., concomitant therapy in cancer).

The number of commercially available products containing probiotics on the market is continuously increasing. Probiotics have been recommended by health care professionals for reasons ranging from their long-term immunomodulatory effects to proven benefits in the management of different health conditions. For probiotic products, irrespective of whether they are classified as food, dietary supplement, functional food, medical food, or drug, there are several important aspects that determine the success rate of a probiotic formulation development from bench to market. The aim of this review is to explore how the current knowledge on microbe–microbe and host–microbe interactions can be used to develop high-quality, evidence-based probiotic formulations, with a focus on the selection of safe strains with relevant functional properties, and the development of probiotic dietary supplements. In addition, we will highlight important aspects of the probiotic manufacturing process that need to be considered during product development and subsequent manufacturing processes to guarantee consistent efficacy and safety of a probiotic product.

## 2. Probiotic Strain Selection and Development

### 2.1. The Concept of Evidence-Based, Indication-Specific and Multispecies Probiotics

There is a wide range of mechanisms of action by which probiotic microorganisms can confer their biochemical, physiological, and thus clinical effects. Unravelling new and precise (molecular) mechanisms of action is currently an active field of research and therefore this is a topic that is regularly reviewed as well [6,7]. Some probiotic activities are directly beneficial for the host, because their beneficial effects are directly mediated through their metabolites. In contrast, other activities require a cascade (i.e., signaling pathways) of (chemical) reactions to finally result in a health benefit for the host. The main mechanisms of action of probiotics include, but are not limited to, activities that directly impact microbe–microbe interactions (e.g., cross-feeding activities, competition for nutrients, production of antimicrobial substances, and production of quorum sensing molecules), or activities that impact host–microbe interactions (e.g., regulation of intestinal barrier permeability, levels of cytoprotective compounds (e.g., defensins) by host cells, host mucus secretion, and gut motility). These mechanisms also include the impact that probiotics can have on even more complex interactions such as the interactions microorganisms can have with our immune system (e.g., promote host defense by priming and conditioning specific adaptive immune responses), the endocrine system (e.g., by influencing hormone levels), the circulatory system (e.g., by influencing systolic blood pressure), or the neurological system (e.g., by influencing neurotransmitter levels). Despite the fact that we just started to understand probiotic mechanisms of action, and further research to elucidate the precise molecular mechanisms of action is certainly still warranted, the knowledge that is already out there can be used as a starting point for the selection of strains with relevant functional properties.

The choice of which functional properties are of interest depends on the health condition that is targeted (i.e., the desired effectivity). Mechanistic understanding of the impact of (certain) microorganisms on physiological processes can be translated into strain selection criteria for a probiotic formulation that is designed to target specific health conditions where these physiological processes are disturbed. Most health conditions that are targeted by probiotics are multifactorial, and as such multiple physiological processes are disturbed. Given the enormous variety of microbe–microbe and host–microbe interactions that can take place, this means that there are numerous ways that microorganisms maintain healthy or positively influence disturbed physiological processes. As there is no such thing as one strain which is able to perform all the beneficial activities by itself, different probiotic strains with different capacities have to be combined to target multiple disturbed processes. There is a large variety in microbial species and strains. Biochemical and physiological properties of probiotics are heterogenous and strain specific. The properties of the different species vary amongst each other and even bacterial strains of the same species can have different properties. For example, some strains of *Lactobacillus rhamnosus* are able to produce mucus-binding pili while other *L. rhamnosus* strains are not [8]. In order to target multifactorial conditions, the probiotic product should consist of a combination of multiple strains, each with its own unique set of properties. Consequently, this means that the final probiotic formulation will often contain strains belonging to different species, thus resulting in a multispecies formulation.

It is important to select probiotics strains that can influence the disturbed physiological processes in the health condition that is targeted by the respective probiotic product. As an illustration, for a product aimed to prevent type 2 diabetes, other strains will be selected than for a product aimed to prevent allergic disease in children, as other physiological processes are disturbed. This can be referred to as “indication specific”. Thus, to design the most effective probiotic formulation for a specific indication, first the disturbed physiological processes in the respective health condition (indication) have to be identified. Next, microbial strains need to be selected that are believed to be able to influence these disturbances. The properties of strains can be studied using a wide range of screening approaches. This knowledge, in combination with insights from scientific literature, can be used as an evidence-based set of selection criteria for the selection of strains that have unique functional properties and can exert specific health-promoting effects and thus can be included in indication-specific and multispecies probiotic formulations.

### 2.2. Characterization and Functional Screening of (Candidate) Probiotic Strains

The strains to be included in a probiotic formulation need to be carefully selected based on their individual characteristics. A wide range of screening approaches can be deployed to screen a collection of (candidate) probiotic strains and to evaluate which strains meet the specified strain selection criteria. These screening models range from simple cell-based in vitro assays to complex ex vivo or animal models [9,10]. While on paper in vivo clinical studies may be most appropriate for testing the effect of microorganisms on the host as they are the closest to real-life situations, in most cases, they cannot be used for high-throughput screening due to their high costs and due to ethical reasons. In addition, models allow researchers to study and get mechanistic understanding of health and disease states in ways that would be inaccessible in a human individual. Each model has its own advantages and disadvantages which have to be carefully weighted before the selection of an appropriate model can be made. For all models, it should be realized that in vitro does not always translate to in vivo efficacy (all models are wrong, but some are useful). However, the development of sophisticated in vitro and ex vivo model systems is advancing rapidly, making them more and more suitable to predetermine or document probiotic properties. Given the diversity of this research field, we will highlight some specific examples of screenings models used to study probiotic–pathogen interactions focusing on the well-known pathogen *Escherichia coli*. Key outcomes of these assays are insights into the mechanisms probiotic bacteria utilize to inhibit, in this case, *E. coli*. Postulated anti-pathogenic strategies of probiotic bacteria include but are not limited to growth inhibition, production of antimicrobial metabolites such as lactic acid, interference with pathogen adhesion by exclusion, competition and displacement, co-aggregation with pathogens, and stimulation of host immune defense against pathogens [11]. Importantly, because these mechanisms are highly species or strain specific, outcomes generated with a specific probiotic strain or species cannot be generalized to all probiotics [12]. Ultimately, these insights contribute to our mechanistic understanding of health and disease and aid better probiotic formulation development.

There is a large range of in vitro assays that are being used for the screening of (candidate) probiotic strains. These in vitro assays are used because of their simplicity and their relative low costs. An important advantage of in vitro assays is their ease of use when screening multiple strains simultaneously. Appropriate in vitro tests have been adopted to select strains based on their ability to survive transit through the different compartments of the gastrointestinal tract. In addition, in vitro assays can be used to study microbe–microbe interactions. These models can also be more complex, such as modelling the complex microbial ecosystems in vitro while ranging from short-term batch incubations to multicompartmental continuous systems. Growth inhibition of *pathogens* by probiotic strains can be assessed using agar-based co-culture methods. For example, growth of *E. coli* DSM 1103 was inhibited by *Lactobacillus rhamnosus* IMC501 and *Lactobacillus paracasei* IMC502 compared to control using both modified cross-streak and radial streak method [13]. Subsequently, an inhibition zone was also detected using cell-free supernatant by agar well diffusion method, indicating a role of acidic pH or other antimicrobial metabolites. Several *Lactobacillus* species have been shown to produce biochemically active compounds against pathogens. For example, the vaginal isolate *Lactobacillus acidophilus* CRL1259 has been shown to inhibit the growth of uropathogenic *E. coli* (UPEC) through the effect of lactic acid production [14]. The antimicrobial activity of probiotic strains against *E. coli* has also been demonstrated using more complex in vitro assays: as a first example, antipathogenic activity of *L. rhamnosus* GG and *S. cerevisiae boulardii* CNCM-I-1079 against enterotoxigenic *E. coli* LMG2092 has been demonstrated using short-term colonic microbiota batch incubations [15]. These incubations adequately simulate the native microbiota and environmental conditions of the proximal colon of the donors, including the colonic mucosal layer. To account for interindividual differences, two different donors, an adult and a toddler, were used as a source of colonic background community. After inoculation of ETEC under simulated dysbiotic conditions, a 40% and 46% reduction in ETEC concentration and a 57% and 46% reduction in ETEC toxin levels were observed upon addition of both strains during the experiments with the adult and toddler donor, respectively. In a second example, the M-SHIME model was used to demonstrate the ability of probiotic *Lactobacillus reuteri* 1063 to decrease mucosal colonization of the adherent invasive E. coli (AIEC) pathogen [16]. This dynamic in vitro gut model allows studying both the luminal and mucosal intestinal microbiota. *L. reuteri* 1063 specifically lowered AIEC numbers in the simulated mucosal environment of the M-SHIME, while they did not affect AIEC numbers in the luminal content. As a possible explanation, *L. reuteri* 1063 treatment increased lactobacilli levels in mucus, leading to antimicrobial effects against AIEC in mucus.

Furthermore, in vitro assays are used to study host–microbe interactions. Examples of in vitro assays are the use of human cell line assays to study bacterial adhesion [17]. Adhesion is also a mechanism by which probiotics can inhibit pathogens (e.g., by exclusion, competition and displacement mechanisms in which presumably steric hindrance and/or competition for similar host receptors may play a role). For example, specific *Lactobacillus jensenii* and *Lactobacillus gasseri* strains have been shown to inhibit adhesion of UPEC to HeLa cells [18] and it has been demonstrated that certain strains of *Lactobacillus crispatus* can exclude UPEC from adhering to vaginal epithelial cells [19]. Further, *L. rhamnosus* GG was shown to partially impair ETEC adhesion to intestinal epithelial cells in vitro [20]. Cell lines such as these are also used to study the effect of microorganisms on epithelial barrier function. In probiotic research, in vitro co-culture assays are also often used to study the interaction with the immune system, as modulation of host immunity is one of the most commonly proposed health benefits attributed to the consumption of probiotics.

Ex vivo models are models cultured outside of an organism and contain functional live tissues with complex cellular environments found in vivo. An example of an ex vivo model that is frequently used in probiotic research is the Ussing chamber [21,22]. This model, in which a small piece of live mammalian tissue or cultured cells can be mounted, is a powerful ex vivo tool for studying permeability or transport across a cell layer. Recent advantages in ex vivo models have led to the development of new promising models such as organoids. Organoids are created from stem cells, and one of their biggest advantage is that they contain different cell types and that they can, for example, recapitulate the normal epithelium and its translatability. New innovative models that are also starting to make their way into probiotic research are organ-on-a-chip models. Organ-on-a-chip models utilize microfluidics, which is a recent advancement in bioengineering, and it shows great potential to mimic complex multiorgan or multilayer systems found in vivo [23]. A human gut-on-a-chip microdevice was used to demonstrate that a probiotic strain can suppress villus injury induced by a specific pathogen [24]. In this study, the gut-on-a-chip model was first colonized with a multispecies probiotic followed by the addition of immune cells (PBMCs), enteroinvasive *E. coli* (EIEC), or both in combination. The injury induced by EIEC infection was significantly reduced when the multispecies probiotic was co-cultured in the lumen of the epithelial channel, as evidenced by the maintenance of high TEER values and the retention of normal villi morphology. Despite their potential, there are still many disadvantages for all these new more complex in vitro models as they are technically challenging, not (yet) suitable for high-throughput screening, and working with live microorganisms in these model systems is still quite challenging.

Our current understanding of the mechanisms underpinning host–gut microbiome interactions is largely derived from animal studies, particularly rodent studies. However, these models are not really practical for the screening of multiple strains, let alone large strain collections. In addition, the relevance of these models to human host–gut microbiome interactions has been questioned [25]. However, in some cases, animal models can be deployed to bridge the gap between the lab and the clinic [26], or preclinical trials using specific animal models can be demanded by regulatory authorities.

In addition to screening the functional properties of probiotic strains using in vitro, ex vivo or animal models, some probiotic functionalities can also be assessed on the genomic level. With the decreasing costs of sequencing and improved algorithms to analyze genomes, in silico analysis has become a powerful tool to screen probiotic strains for their potential functional capabilities. The application of in silico functional screening can thus be a powerful approach to preselect strains for subsequent in vitro, ex vivo or in vivo assays. Conventional in silico screening requires the nucleotide or amino acid sequence for a gene or protein of interest to be compared with the genomes of the probiotic candidates. Such homology-based screening requires that the genetic basis (i.e., coding sequence) of a functionality is known. In silico analysis can be a powerful strategy when one wants to screen strains for their capability to synthesize certain well-characterized metabolites. For instance, the genes required for the bacterial production of short-chain fatty acids or amino acids, which are all metabolites that are important for human health, are well described and thereby make them ideal candidates for preceding in silico analysis. However, for functionalities where the precise genetic basis remains unknown, such a conventional in silico approach is not suitable. Especially for complex functional traits (such as immunomodulation), the genetic basis has most often not (yet) been unraveled, as they require the interplay and expression of a wide range of genes from both microbial strains and their host. Obviously, the rapid advancement of research related to functional genomics will generate more knowledge about the genetic basis of certain functionalities, which then can be screened with conventional in silico methods. An example is provided by the recent study published by Kenny et al. in which the authors were able to predict and validate a new group of microbial enzymes involved in cholesterol metabolism [27]. A multidisciplinary strategy was used combining existing metagenomics and metabolomic datasets, microbial genome mining, and in vitro biochemical and culture-based assays. Using this approach, the authors were able to identify the specific genes responsible for the biochemical conversions and link this property to a specific clade of, in this case, yet uncultured, bacterial species. Due to increased computational power and smarter algorithms, it is theoretically also possible to apply sophisticated artificial intelligence algorithms to predict complex functional traits of microorganisms. A current disadvantage is that those algorithms need to be fed with large amounts of genotypic and phenotypic data to gain accurate predictions, which are currently not yet available for complex functional traits. However, such algorithms are, for example, already used to predict antimicrobial resistance in pathogenic bacteria [28] or to stratify diseased from healthy individuals based on microbiome composition data [29]. It will be a matter of time before such methods can reliably be used to predict complex functionality traits of microorganisms.

### 2.3. Identification and Safety Assessment of (Candidate) Probiotic Strains

Before deliberately exposing humans to large quantities of microorganisms, there is a moral but also a legal obligation to ensure their safety. In the European Union (EU), for example, food safety and thus also the introduction of microorganisms into the food chain is covered by EU regulation (EC) No 178/2002. Several research groups, organizations and authorities have attempted to provide guidelines and/or recommendations for determining the safety of microorganisms intended for use by humans. Although there is still a lack of consensus on the methodology to assess safety of microorganisms, there is general acceptance on several aspects of the safety evaluation.

There is consensus that for establishing the safety profile of a strain, unequivocal taxonomic identification should be performed. Therefore, the identity and taxonomic position of the candidate probiotic strain(s) need to be established using state-of-the-art methods in molecular biology, biochemistry and physiology. Throughout the years, different approaches have been used to identify and classify organisms [30,31,32]. Classical approaches make use of morphological, physiological and metabolic characteristics of organisms. With this approach, microbes are grouped based on easily-observed phenotypic characteristics (e.g., cell morphology, Gram staining, motility, and structural features) and on distinguishing physiological features (e.g., carbon source utilization patterns, and growth characteristics). Following this approach, the phenotypic characteristics of the strain of interest are compared to those of the type strains of related species. Since analytical tools have been available for characterization of biochemical properties of cells, microbes are also being grouped based on chemotaxonomic characteristics such as cellular fatty acid or polar lipid composition. Advances in molecular techniques, fortunately, provide an important contribution to definitely identifying and classifying microorganisms on the basis of their genotypic characteristics. For the past decades, 16S rRNA gene sequence analysis has been the gold standard for bacterial identification and taxonomic classification. However, one noteworthy limitation of identification based on this marker gene is that for some bacterial species, 16s rRNA gene sequences do not provide enough resolution and therefore they not always allow for species-level identification and thus also not for taxonomic classification. Further, as 16S rRNA gene sequence analysis is based on a single gene, it is by no means suitable for strain-level identification. As studies have shown that probiotic activities can be strain specific [33], microbial identification at the strain level becomes mandatory. Hence, whole-genome sequence-based analyses are gaining foothold as the standard method for strain identification. As whole-genome sequencing has become affordable, the gold standard for taxonomic classification is now also shifting toward whole-genome sequence-based analysis. For taxonomic classification, genome-based phylogenetic trees are generated to establish the taxonomic position of the strain of interest relative to other (publicly available) genomes of the respective and related species. However, it has to be recognized that the current microbial classification is shaped by historic reasoning. As it is generally acknowledged that classification should rest on the highest quality of data, classification is continuously revisited as we gather more molecular data and taxonomic assignments have to be adjusted accordingly. Classification changes can lead to name changes for strains. A recent example for this, that has impacted the probiotic field, was the reclassification of the genus *Lactobacillus*, leading to new genus names for many well-known probiotic strains [34]. Although the basis for taxonomic changes are scientifically valid, changes such as these do provide many communication challenges for both science and industry and therefore it will take time before name changes such as these are fully adopted by all stakeholders in the probiotic field.

Following detailed strain identification, a thorough safety assessment should be performed. In scientific literature, the safety considerations related to human consumption of live microorganisms are topics that are regularly reviewed [35,36,37,38,39,40,41,42] It is generally agreed that safety of microbial strains depends on the intrinsic biochemical and physiological nature of the respective microorganism. A brief summary follows; live microorganisms (including commensal) may be responsible for the following adverse effects: (1) production of metabolites that are deleterious to the host (toxigenicity), (2) causing opportunistic (systemic) infections (pathogenicity), (3) stimulating an excessive immune response in susceptible individuals, and (4) transferring genes to other microorganisms (e.g., antimicrobial resistance genes). Based on these safety concerns, it is generally recommended that before a microorganism is used in its intended human target population(s), the microorganism has been properly characterized and checked for absence of transferable antimicrobial resistance genes as well as absence of toxic and/or pathogenic properties. Furthermore, safety depends on the intended use of the microorganism, the mode of administration, level of exposure (dose, duration and frequency), and the health status of the consumer population (e.g., healthy young adults, elderly, and immunocompromised patients).

For safety assessments of microorganisms using genomic information, (draft) guidelines have been provided by agencies such as the European Food Safety Authority (EFSA) [43,44]. These guidelines provide recommendations on specific databases that can be used for genome comparative analyses, such as the Virulence Factor DataBase (VFDB) [45]. Further, more importantly, they provide guidance on when one should consider and report a virulence gene to be present (including cut-off values for percent sequence coverage and percent sequence similarity). The presence of genes encoding virulence factors may trigger further phenotypic testing (e.g., cytotoxicity tests). If the strain under evaluation belongs to a taxonomic group that contains known mammalian toxin producer(s), it must be examined for toxin production. Further, if the strain under evaluation belongs to a group with known hemolytic potential, determination of hemolytic activity is required. In addition to assessing virulence factors, thorough assessment for certain deleterious metabolic activities (e.g., production of biogenic amines) is deemed essential. Once phenotypic testing confirms that a strain has certain virulence properties or deleterious metabolic activities, one should reconsider the suitability of that particular strain as a candidate probiotic strain.

In addition to the possible direct detrimental effects the deliberately administered microorganisms can have on human health, there is general consensus that the deliberate introduction of new microorganisms to an existing ecosystem increases the risk of spreading antimicrobial resistances. This is seen as a general health concern. Therefore, the antimicrobial resistance profile of the candidate probiotic strain(s) needs to be assessed, complemented with an assessment of the strain’s potential to transfer antimicrobial resistance. The strains need to be phenotypically screened for antimicrobial resistances, and per antimicrobial of interest, the resulting measured minimum inhibitory concentration (MIC) should be compared to the cut-off values set for that particular species or microbial group. Further, for these analyses, guidelines have been provided, for example, by the EFSA who has provided cut-off values for specific species or microbial groups that are currently added to the food and feed chain [43,46]. A genome analysis should be performed when MIC values are found that are above those cut-off values, in order to examine the genetic basis of the antimicrobial resistance. The first step is to specifically search for genes that convey antimicrobial resistance. Further, for these analyses, separate (draft) guidelines, as for example those provided by the EFSA [43,44], provide recommendations on specific databases that can be used for these comparative analyses. For these analyses, it has been recommended to use at least two databases, such as CARD [47] and ARG-ANNOT [48]. Further, in this case, guidelines provide recommendations on when one should consider and report a gene as present (including cut-off values for percent sequence coverage and percent sequence similarity). Once the presence of antimicrobial resistance genes has been confirmed, the next step is to assess the likelihood that these specific antimicrobial resistances can be transferred to other microorganisms. Horizontal gene transfer is the primary mechanism facilitating the exchange of genetic information between microorganisms. Horizontal gene transfer enables the acquisition of antimicrobial resistance genes by microorganisms and thereby facilitates the spread of resistance genes. Mobile genetic elements play a key role in horizontal gene transfer and those elements include those that are able to move within or between DNA molecules (e.g., insertion sequences, transposons, and gene cassettes/integrons), and those that are able to transfer between bacterial cells (e.g., plasmids and integrative conjugative elements). Therefore, the localization of the antimicrobial resistance gene is an important step to take once antimicrobial resistance genes have been identified [49]. Once an antimicrobial resistance is flagged as potentially transferable, the strain should no longer be considered as a candidate probiotic strain.

In general, a history of safe use in the human population provides a strong safety profile to a probiotic strain at start. A standardized inventory of microorganisms with documented histories of safe use in food is maintained by the International Dairy Federation (IDF) in collaboration with the European Food and Feed Cultures Association (EFFCA) since 2002 [50,51]. This inventory contains a species-level overview of microorganisms used by the food industry in food to a significant degree, and those that have a long history of safe use in food without any adverse effects. In the European Union (EU), the EFSA introduced the Qualified Presumption of Safety (QPS) approach to facilitate and simplify safety assessment of microorganisms that require premarket authorization [50]. This entails that all strains belonging to a certain (sub-) species, can a priori be considered safe in several or most aspects, and only specified remaining safety aspects require assessment at the strain level. This includes the examination of the absence of virulence factors and/or specific toxic metabolites that are of concern for certain taxa (e.g., for *Bacillus* spp.), and absence of transmissible antimicrobial resistances for all microorganisms. In the EU, a QPS status is not a mandatory regulatory requirement for live microorganisms used in foods. However, worldwide, there are conceptual differences in the exact details of safety assessment needed for live microorganisms that are included as dietary ingredients in foods. For example, in the United States, products are regulated by the Food and Drug Administration (FDA). The FDA introduced the ‘Generally Recognized as Safe’ (GRAS) concept for food and substances (including microorganisms) used in conventional foods [52]. For microorganisms, GRAS assessments are performed at the strain level and the assessments are limited to its intended conditions of use. In conclusion, the safety assessment of a candidate probiotic strain follows a general approach overall. However, the details depend on the intended use and target market of the finished probiotic product.

### 2.4. Strain Manufacturing Process Development

By combining the strain identification and characterization efforts based on functionality and safety profile, strains can be selected that show relevant functional properties and that can be considered safe for human consumption. However, another important hurdle is manufacturability: Promising strains might not make it as successful probiotic strains if they do not make it through the manufacturing process development steps. Beginning with the end in mind, the desired commercial probiotic formulation will consist of viable, concentrated cells that are stable and will have consistent performance in the intended application. Especially for high-quality probiotic formulations with doses established through clinical trials, high cell count and long shelf-life stability in a variety of different temperature and humidity conditions are expected by customers. To control the manufacturing process and thereby the quality of each strain in the final probiotic formulation, it is common practice that the cell mass of each strain is produced separately.

In general, the manufacturing process of microorganisms for probiotic dietary supplements follows a set of common steps, as is also recently reviewed [53]. In summary, the final commercial manufacturing process of the required amounts of viable, concentrated and stable microbial cells comprises upstream processing steps (e.g., media preparation and inoculum preparation), the actual fermentation step during which the cell mass is produced, and downstream processing steps (e.g., cell concentrating and cell drying) in which the cell mass is recovered, concentrated and stabilized. The resulting material can then be used for blending with other ingredients to produce the finished probiotic product. However, before a strain can be successfully produced at a commercial scale, costly and labor-intensive manufacturing process development work has to be performed to get a strain from lab-scale, followed by pilot-scale, up to large-scale commercial production. A lot of small changes in the production of the concentrated probiotic cells can have a large overall effect on product quality and performance. There are several important aspects that need consideration during the strain development process. Already within the fermentation step, there are many variables that need to be evaluated and validated before and during scaling up of the manufacturing process. First of all, the medium composition—specifically the type and amount of carbon source (e.g., glucose), the type and amount of nitrogen source (e.g., yeast extract) and the type and amount of other macro and micronutrients (e.g., magnesium)—has to be established carefully. Furthermore, the conditions during the actual fermentations, such as the starting pH and pH set point during the fermentation, incubation temperature, stirring speed and dissolved oxygen level, require attention. In addition, the growth phase and volume of the inoculum, the amount of fermentation steps, as well as timing of the final harvest (e.g., after depletion of the carbon source) will have an impact on the final quality and performance of the probiotic cells. Already during the strain manufacturing process development, important choices have to be made regarding the intended use and application of the finished probiotic product, as these choices will influence the downstream processing steps. Probiotics, particularly when included in dietary supplements, are generally expected to have up to 24 months of stability at ambient temperature and humidity. One way to preserve viable bacterial cells for a prolonged time is by drying the cells. Drying of microbial cells is usually achieved by freeze-drying or spray-drying. To facilitate cell survival during drying, the cells are often formulated beforehand. The formulation forms a matrix that embeds the cells and protects them during freezing and drying from various detrimental stresses imposed on the cells. If the choice has been made to produce the strain as freeze-dried cells in powder form, many subsequent steps and variables will need to be evaluated and validated before scaling up the production process can take place. For example, centrifugation speed and method, and which cryoprotectants (e.g., mannitol), lyoprotectant (e.g., maltodextrin) and/or other compounds (e.g., buffers) to use for protection during the initial freezing and subsequent freeze-drying step are important aspects to evaluate. Further, the settings of the freeze dryer—specifically the temperature and vacuum profiles for effective primary and secondary drying—are of utmost importance. In summary, manufacturing process development work is costly, labor intensive and requires specialized expertise because each step in the process depends on the output of the previous steps. Furthermore, process upscaling can be very challenging, as certain variables change during scaling up such as volumes and equipment that are used. For example, transfer processes such as gas mass transfer (e.g., O_2_ and CO_2_) and heat transfer processes are often not properly accounted for during upscaling as they do not scale linearly. In addition, for each strain, dedicated manufacturing process development work is needed because of strain-dependent sensitivities to environmental factors.

## 3. Important Aspects to Consider during Product Formulation and Manufacturing Process Development

### 3.1. Product Formulation and Manufacturing Process Development

There are many aspects of product manufacturing, packaging and handling process of a probiotic product that influence the quality of the finished product. During the first stage of product manufacturing, stabilized (e.g., freeze-dried) cells are formulated (blended) with other ingredients such as excipients (bulking agents) and flow aids. To ensure microbial viability and product effectiveness, additional active ingredients can be added to a probiotic formulation to optimize properties such as the viability, stability, metabolic activity and gastrointestinal survivability of the microbial cells. In addition to the intrinsic gastrointestinal survival-related properties of the probiotic strains (e.g., their sensitivity to acid, digestive enzymes or bile), one of the key factors in microbial bacterial survival is to ensure that the microbial cells are as “fit” as possible before they enter the gastrointestinal tract. For example, as a result of long-term storage or incorrect revival from their (freeze-) dried state, microbial cells could have been damaged, which impacts their sensitivity to the environmental conditions during GI passage. The addition of extra ingredients in the formulation (such as minerals and prebiotics) can increase the long-term stability of the microbial cells and ensure their viability during re-activation (rehydration) and thereby increase their metabolic activity and gastrointestinal survivability. In the end, this results in improved product effectiveness. Furthermore, the addition of other functional ingredients such as vitamins or minerals could be desired in light of the intended use of the product to provide additional health benefits. All of these extra ingredients can influence key properties of the final formulation and therefore the impact of these ingredients on the performance of the probiotic cells needs to be evaluated and validated (ingredient compatibility testing). However, unlike other dietary supplements, all probiotic stains are live organisms and their viability and activity can be greatly impacted by the addition of high amounts of additional (functional) ingredients as these compounds are often also involved in subsequent microbial physiological and metabolic processes. In addition, processing and long-term storage conditions may impact probiotic performance as well. Therefore, it is important to pay attention to the final product format and packaging. For probiotic dietary supplements, powder sachets or stick packaging, tablets or capsules are the most commonly produced formats, and usually these products can be stored at ambient conditions for long periods of time. During the product formulation and manufacturing process, reproducibility is important to ensure constant high performance and quality. To ensure this, quality control throughout the whole manufacturing process, from raw materials to the finished product is essential, as is the documentation of adequate quality control.

### 3.2. Quality Properties of Probiotic Formulations

#### 3.2.1. Viability Throughout Shelf Life (Stability)

Probiotics are live microorganisms by definition and therefore one of the most important quality aspects is to keep the probiotic microorganisms viable over time. Therefore, it is essential that the finished product contains viable cells. Viability is usually measured in colony forming units. In addition, for optimal efficacy of the probiotic formulation for the end user, it is important to ensure the viability of the microbial cells until the end of shelf life (stability). Thus, the quantification of post-manufacturing viability is essential to ensure the labelled quantity of viable probiotic cells also at the end of the shelf life. Many different factors may affect the stability of probiotics, including environmental factors such as water activity, temperature, pH, and oxygen exposure. To ensure probiotic viability and stability, attention should be given to product technological aspects such as matrix design, final product format, packaging material, storage conditions, handling and distribution logistics, as all these factors can have a major impact on product viability throughout shelf life.

#### 3.2.2. Gastrointestinal Survival

To survive passage through the stomach and small intestine, probiotic strains must tolerate the hostile conditions they will encounter during their passage through the gastrointestinal tract. Relatively simple in vitro models are used to simulate the passage of the probiotic microorganisms through the gastrointestinal tract and to evaluate their survival. Quantitative extrapolation of in vitro models to probiotic performance in vivo remains challenging. However, these in vitro models are useful to assess specific important (environmental) factors such as exposure to a low pH, digestive enzymes including pepsin, pancreatin, and bile salts. Although gastrointestinal survivability of a strain is largely dependent on the intrinsic physiologic properties of the probiotic strains themselves, choices in additional extra ingredients (e.g., matrix design) and final product format can influence the gastrointestinal survivability of the microbial cells in the finished product.

#### 3.2.3. Activity

Metabolic or biological activity is one of the most important parameters for the quality of a probiotic product. It is even more important than the amount of colony forming units in the product. Due to the addition of extra ingredients to the final formulation or the exposure of the product to certain environmental conditions (for example, the conditions encountered during gastrointestinal passage), the microbial cells can be damaged in such a way they will still survive, but no longer reach their full (metabolic) activity level. These cells will be counted in a viable cell count but will potentially not perform on the mechanisms they were selected for. For most probiotic mechanisms of action, it is imperative that the cells are metabolically active. Each strain in a product has its own functional activities it has been selected for, but there are also a few common probiotic mechanisms that can be used to assess both metabolic activity and probiotic activity at the same time, and thereby these activities can be used indirectly as a read out to assess the overall functional performance of the finished product. One example of an approach to assess metabolic activity of microbial cells is to measure the formation of metabolic end products. As currently most probiotic strains are (facultative) anaerobes, acids are typical metabolic end products. Further, more specifically, as most currently marketed probiotics are lactic acid bacteria, lactic acid as the main metabolic end product is a read out that can be used to evaluate metabolic activity. Thus, it can be stated that the more (lactic) acid probiotic microorganisms produce, the more metabolically active they are. As also discussed in earlier paragraphs, lactic acid is one of the metabolites produced by many probiotics strains by which they can inhibit the growth of pathogens, and it is therefore also an indicator of probiotic activity. Therefore, measuring (lactic) acid production is considered an excellent method for measuring both the metabolic and probiotic activity of probiotic strains. The production of (lactic) acid by the probiotic microorganisms can also be measured over time, for example after a simulation of passage through the stomach (by addition of an acid drop), and can as such be again used as a parameter of metabolic activity of the probiotic product.

### 3.3. Extensive Quality Monitoring Throughout the Manufacturing Process

#### 3.3.1. Viability and Composition Validation

Since probiotics are live microorganisms, a method of quantification to measure cell viability is essential. Traditionally, microbial viability is assessed based on the capacity of microbial cells to replicate to detectable levels, either as a colony on agar or by turbidity in broth culture medium. Up to this date, microbial enumeration by plate count methods is the gold standard method to quantify microorganisms in the probiotic industry, and it is generally accepted that the amount of probiotic microorganisms should be declared in colony forming units (CFU) per gram or serving [54]. Despite its technical simplicity, there are many technical challenges with this approach and alternative methods of measure of viability are currently being developed. In recent years, flow cytometry has found its way into the field of microbiology and has been developed as a simple tool for the rapid analysis of viability. Despite these advances in analytical methods, composition validation remains a complex situation for products containing multiple strains as the available methods often fail to differentiate between different strains. Molecular methods offer another promising alternative to traditional plate count enumeration of probiotic products. Due to the progress in genome sequencing and bioinformatics, this is an approach that has the potential to rapidly and accurately enumerate viable probiotics down to the strain level. Strain-specific polymerase chain reaction (PCR) assays can be developed with the use of strain-specific PCR primer sets and probes that target unique insertions/deletions or single-nucleotide polymorphisms in DNA sequences based on which they are able to even distinguish phylogenetically similar strains. In addition, the field is currently developing methods to quantify viable cells using a PCR-based method in combination with live-dead staining methods [55,56]. These methods are based on the approach that prior to DNA extraction and amplification, samples are treated with a viability dye, a molecule that selectively enters cells with damaged membranes and intercalates into their DNA. As live cells generally exclude the dye, the result is that only their unmodified DNA is selectively amplified by PCR. Currently, there are several DNA-intercalating dyes available that can be applied to develop viability PCR assays. The development of viability PCR assays in conjunction with the development of highly specific (i.e., strain-specific) PCR assays holds great promise for the probiotic industry. The end goal is to be able to evaluate the viability of each individual strain throughout the shelf life of the probiotic product.

#### 3.3.2. Safety and Quality Monitoring

Product safety and quality are of great importance and probiotic manufacturers are advised to choose a quality system that meets product safety and quality requirements. In the context of dietary supplements, there are food safety management systems that provide a systematic approach to controlling food safety hazards within a food business. In the EU, food business operators are, for example, required by regulation (EC) No 852/2004 (that provides general rules on the hygiene of foodstuffs) to work with a food safety management system based on Hazard Analysis Critical Control Point (HACCP) principles. Hygiene is an important point of attention throughout manufacturing, as well as the control of the raw materials and the logistics process. In addition, certain required activities in the manufacturing, packaging, labelling and storage of the dietary supplements are needed to ensure that a dietary supplement complies with its labelling and do not contain harmful or unwanted substances such as microbial contaminants, pesticides, heavy metals or other impurities. By the development, implementation and maintenance of a food management system, food safety is addressed through the analysis and control of biological, chemical, and physical hazards from raw material production, procurement and handling, to manufacturing, distribution and consumption of the finished product. There are recent reviews that provide an excellent overview of the quality control and quality assurance aspects that need to be considered during the manufacturing of probiotic dietary supplements in order to comply with regulatory guidance and industry standards [53,54]. Recommendations include performing certification by independent third-party organizations and following standards established or required by government and non-governmental agencies, such as the FDA, the EFSA, and Codex Alimentarius. Measures such as these provide assurance of quality and facilitate regulatory compliance of an ingredient or finished product.

## 4. Clinical Efficacy of a Probiotic Product

Despite the great efforts that often go into the development of a probiotic product, clinical efficacy cannot be predicted. Even though the aforementioned aspects of in silico screenings, in vitro assays and regulatory obligations are becoming better, more advanced, and stricter, clinical testing of the finished product in order to prove its efficacy remains inevitable. Currently, probiotics are the subject of extensive studies in health and medical research as their potential indications are steadily increasing due to the growing understanding of the impact of the (gut) microbiota on human health. The investigated indications vary greatly and range from relieving gastrointestinal discomfort (most commonly AAD) to modulation of the gut–brain axis. Taking the previous paragraphs into account, it is important that the clinical studies are performed with the probiotic formulation (and form) as available on the shelves to ensure that the end consumer can expect the same beneficial effects as demonstrated in the respective clinical study. The following paragraphs discuss some of the main indications and highlight a few examples of available clinical trials in which probiotic formulations showed clinical efficacy. In this context, we also recommend the excellent review papers by Ritchie et al. [57], Zhao et al. [58] and Huang et al. [59] that provide systematic overviews of the current available data of clinical efficacy in regard to the respective indications.

### 4.1. Antibiotic-Associated Diarrhea

The use of probiotics to treat AAD is among the most well-established indications. A study published in 2008 in the American Journal of Gastroenterology investigated the probiotic effects on gut microbiota composition in healthy volunteers during and after amoxycillin intake using a multispecies probiotic formulation [60]. The investigation clearly showed that the use of antibiotics leads to a destabilization of gut microbiota and can be an indirect cause of diarrhea. Probiotic intervention led to a significant improvement in microbiome composition and microbiome diversity. The probiotic intervention decreased incidence of AAD (and presumably also reduced associated health care costs) irrespective whether the probiotic was taken during or after antibiotic treatment.

One particular pathogen that is often associated with AAD is *Clostridioides difficile* (formerly known as *Clostridium difficile*). The severity of diarrhea induced by this very pathogen can differ from mild cases to pseudomembranous colitis. A study conducted by Hell et al. showed that the same probiotic formulation as mentioned above led to a significant reduction in *C. difficile* infections during Vancomycin intake. This effect appeared sustainable, as also, after 6 months post-intervention, *C. difficile* could not be detected in stool samples [61]. Another study that investigated the efficacy of probiotics in regard to AAD was recently conducted in selected Dutch nursing homes: The study had a pragmatic participatory evaluation design and included residents with somatic and/or psychogeriatric conditions frequently taking antibiotics. Ninety-three residents provided data on 167 episodes of antibiotics use, of which 84 episodes included supplementation with probiotics and of which 83 episodes did not include probiotics supplementation. The number of episodes with AAD when using probiotics was significantly lower than when no probiotics were used (20% vs. 36%; *p* = 0.022, Chi-square) [62].

### 4.2. The Infantile Microbiome and Its Effect on the Maturation of the Immune System

Immunological studies have long suggested the importance of gut microbiota on the maturation of the infantile immune system. As probiotic bacteria possess immunomodulating effects, their impact on the mammalian immune system was one of the first indications that was investigated when clinical probiotic research gained momentum. To date, there are promising studies on specific probiotics to be used during pregnancy in order to reduce the risk of allergies, asthma and atopic eczema in children. An example of a clinical randomized controlled trial in this context is the PANDA study from 2009 [63]. The trial resulted in a preventive effect on the incidence of eczema in high-risk children by a multispecies probiotic formulation, which seemed to be sustained during the first 2 years of life. In addition, similar trials showed that the administration of the same probiotic formulation to the mother in the last 8 weeks of pregnancy and to the baby during the first months of life results in a significant reduction in the incidence of 3 month colics [64].

### 4.3. Metabolic Diseases

Probiotic research is currently highly focused on treating metabolic diseases such as obesity and type 2 diabetes. The idea behind this concept is the reduction in endotoxin-induced metabolic disease (due to intestinal hyperpermeability). Since specific probiotics are evidently able to strengthen the intestinal barrier, their use in the reduction in circulating endotoxin (i.e., lipopolysaccharide (LPS)) levels has gained significant interest in recent years. A placebo-controlled randomized trial tested the effect of a multispecies probiotic formulation on glucose metabolism, lipid profile, subcutaneous fat, uric acid levels and LPS concentration in serum of obese, post-menopausal women—and finally the clinically relevant Homeostatic Model Assessment for Insulin Resistance (HOMA-IR) score [65]. Significant positive results were achieved in all the mentioned parameters. Similar results were found in another clinical placebo-controlled randomized trial, showing a statistically significant improvement in insulin resistance and abdominal obesity after intake of the very same probiotic formulation [66]. This particular pair of independent studies is seen as an important hallmark in probiotic research as it demonstrates that there is reproduceable evidence within the clinical setting. Thus, the use of multispecies probiotics should be considered as an important evidence-based adjuvant therapy in metabolic diseases.

### 4.4. Probiotic Impact on the Gut–Brain Axis

The gut microbiota plays an important role not only in gastrointestinal function but also in the regulation of mood, anxiety and cognition via bidirectional communication with the brain via the vagus nerve and/or the biochemical flux of small molecules from the colon to the brain. So far, studies examining the clinical relevance of the gut-brain axis provide multidimensional evidence that administration of a multispecies probiotic formulation and the associated changes in gut microbiota composition have a significant interrelated impact on subject behavior and emotional memory performance [67,68]. Additional studies investigating the same multispecies probiotic revealed a positive impact on cognitive function and neuroinflammation in patients with psychiatric [69] and neurodegenerative [70] diseases. Further evidence is provided by several trials performed with another multispecies probiotic formulation. These studies showed positive impact on mood and depressive-like behavior in rats and demonstrated protection against the depressive-like behavior-promoting effects of a high-fat diet [71,72]. Evidence is further substantiated by the same formulation showing positive effects on cognitive reactivity to sad mood in healthy subjects [73] and mild to moderately depressed patients [74] as well as demonstrating a significant increase in working memory performance during stress [75]. While the gut–brain axis and its probiotic modulation is still a relatively young field of research, initial RCT studies show promising results. There is yet a lot of open questions to be answered, particularly within the context of molecular gut–brain causalities.

### 4.5. Liver Diseases

Continuously growing knowledge on the characteristics of various microbial strains (e.g., strengthening of the intestinal barrier and breakdown of LPS) has led to the development of probiotic formulations that aim to protect the liver by exercising a positive influence on compromised liver function in patients with liver cirrhosis via the modulation of the intestinal microbiome. As an example, a clinical study conducted by Horvath et al. [76] showed that a stabilization and even improvement in liver function in patients with liver cirrhosis can be achieved through the administration of a multispecies probiotic formulation in a considerably high dosage of 1.5 × 10^10^ of viable cells. While the present state of studies in this context is still scarce, the aforementioned positive effects of probiotics on metabolic disease and circulating LPS levels portend a significant clinical potential in treating liver disease, as the underlying molecular pathogenesis is similar, being the elevated influx of pro-inflammatory metabolites from the intestine to the liver and the circulation.

### 4.6. Conclusions and Outlook

**Figure 1:**
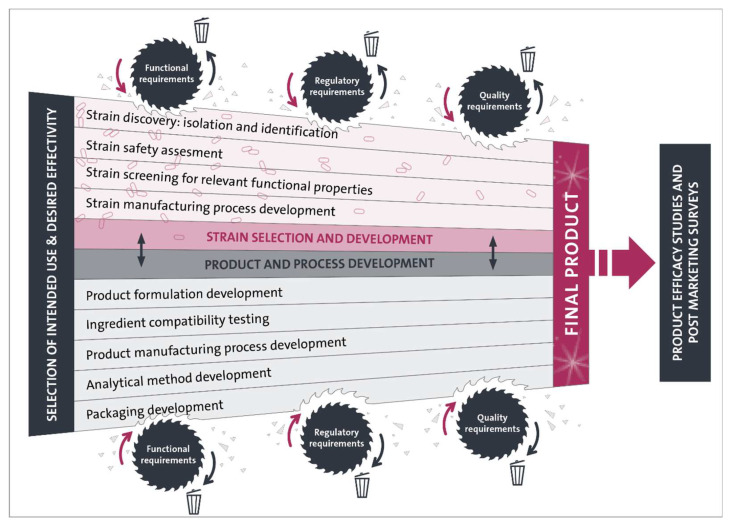
Schematic overview of the multidisciplinary approach that is needed for the development of an evidence-based, indication-specific, multispecies and ready-to-market probiotic. All the steps needed for strain and product development are shaped by the functional, regulatory and quality requirements of the final product. During this process, several candidates (of strains, additives, production process blueprints, and packaging ideas) get discarded. In addition, manufacturing processes are finetuned and analytical methods need to be developed to guarantee consistent efficacy and safety of a finished probiotic product. Strain and product development is followed by product efficacy studies and post-marketing surveys in order to demonstrate clinical efficacy of the finished probiotic product.

As discussed in this review and summarized in Figure 1, there are many important aspects that need to be evaluated during the development of an evidence-based, indication-specific, multispecies and ready-to-market probiotic. Careful consideration of all these aspects will increase the success rate of the probiotic development from bench to market. The evident assurance of quality, purity and strength of probiotic dietary supplements is of utmost importance, as health care professionals and end consumers need to be able to trust that theoretical strain properties are also actually executed once the probiotic microorganisms reach their site of action, which is currently most often the human intestine. While the number of probiotics currently available on the market is high (and steadily increasing), clinically randomized trials that actually prove their respective efficacy remain scarce. Nevertheless, as discussed in the review, there are multispecies probiotic formulations that have already shown efficacy in numerous clinical trials and in a wide array of health conditions. In the coming years we expect that probiotic research continues to concentrate on the development of indication-specific probiotic dietary supplements. Those will have the potential to be used as a concomitant therapy along conventional medical treatments without causing side effects or requiring a physiological adaptation phase. With new scientific insights, we foresee that the development of science-based, multispecies probiotics for a wide range of new medical indications not discussed in this paper will increase as well. In addition, as diagnostic and predictive tools are rapidly evolving, we anticipate that probiotics will increase their foothold in personalized health care, tailoring food-based and medical therapies towards the needs of each individual patient. More uniformity and standards in terms of safety, quality and efficacy determinants will also strengthen the position of probiotic dietary supplements and thus expand the global market of probiotics, which will in turn increase the need for more (clinical) studies in order to establish those determinants in practice. All these developments will also add to the ongoing debate on regulatory classification of probiotics. The outcome of these debates will also influence and sharpen the functional, regulatory and quality requirements that need to be evaluated and validated during the development of a successful high-quality and ready-to-market probiotic.
